# Extracellular vesicle-encapsulated microRNA-23a from dorsal root ganglia neurons binds to A20 and promotes inflammatory macrophage polarization following peripheral nerve injury

**DOI:** 10.18632/aging.202532

**Published:** 2021-02-17

**Authors:** Yamei Zhang, Junying Liu, Xin Wang, Jinfeng Zhang, Chenchen Xie

**Affiliations:** 1 Sichuan Medicine Key Laboratory of Clinical Genetics/Central Laboratory, Affiliated Hospital of Chengdu University, Chengdu 610081, P.R. China; 2 Department of Pediatrics, Affiliated Hospital of Chengdu University, Chengdu 610081, P.R. China; 3 Department of Neurology, Affiliated Hospital of Chengdu University, Chengdu 610081, P.R. China

**Keywords:** peripheral nerve injury, extracellular vesicles, microRNA-23a, A20, sensory neuron

## Abstract

Extracellular vesicles (EVs) are capable of transferring microRNAs (miRNAs or miRs) between two different types of cells and also serve as vehicles for delivery of therapeutic molecules. After peripheral nerve injury, abnormal expression patterns of miRNAs have been observed in dorsal root ganglia (DRG) sensory neurons. We hypothesized that sensory neurons secrete miRs-containing EVs to communicate with macrophages. We demonstrated that miR-23a was upregulated in DRG neurons in spared nerve injury (SNI) mouse models. We also found that miR-23a was enriched in EVs released by cultured DRG neurons following capsaicin treatment. miR-23a-containing EVs were taken up into macrophages in which increased intracellular miR-23a promoted pro-inflammatory phenotype. A20 was verified as a target gene of miR-23a. Moreover, intrathecal delivery of EVs-miR-23a antagomir attenuated neuropathic hypersensitivity and reduced the number of M1 macrophages in injured DRGs by targeting A20. In conclusion, these results demonstrate that sensory neurons transfer EVs-encapsulated miR-23a to activate M1 macrophages and enhance neuropathic pain following the peripheral nerve injury. The study highlighted a new therapeutic approach to alleviate chronic neuropathic pain after nerve trauma by targeting detrimental miRNA in sensory neurons.

## INTRODUCTION

Peripheral nervous system injury commonly leads to neuropathic pain, which is often marked by spontaneous pain and allodynia [1]. Pain is induced upon the detection of noxious stimuli at terminals of dorsal root ganglia (DRG) neurons innervated in the peripheral nervous system [2]. It is reported that spared nerve injury (SNI)-induced neuropathic pain contributes to an enhanced M1 polarization and secretion of inflammatory factors in the prefrontal cortex [3]. Macrophages may exhibit pro-inflammatory or anti-inflammatory roles in response to injury, and their activated states are known as classical activation (M1) and alternative activation (M2). So far, few studies have explored the role of macrophage polarization in SNI models of neuropathic pain [4].

Extracellular vesicles (EVs) are nanometer-scale particles and consisted of exosomes, microvesicles, and apoptotic bodies; they are involved in diverse biological processes by delivering effectors including transcription factors, oncogenes, small and large non-coding regulatory RNAs (such as microRNAs (miRNAs or miRs), messenger RNAs (mRNAs) and infectious particles into recipient cells [5]. miRNAs refer to small non-coding RNA molecules, which serve as important regulators of mRNA translation or mRNA stability by directly binding to the 3’-untranslated region (3’-UTR) of the target mRNAs [6, 7]. In addition, the effects of miRNAs on neuropathic pain have been demonstrated with increased clinical potential [8]. Karl et al. reported that miR-21 serves as a therapy target for SNI in B7-H1 KO mice [9]. Epigenetic inhibition of miR-23a has the potential to serve as a potential novel target for therapeutic intervention of peripheral nerve injury-induced nociceptive hypersensitivity [10]. The role of miR-23a in reducing hypoxia-induced neuronal apoptosis has also been documented in a previous study [11], while its effect on SNI-induced neuropathic pain has not been investigated. miR-23a has been demonstrated to regulate the polarization of macrophages and induce the release of pro-inflammatory cytokines by targeting A20 and the nuclear factor κB (NF-κB) signaling during cancer progression [12]. A20 (also known as TNFAIP3), a potential anti-inflammatory element, is involved in the pathogenesis of multiple inflammation-related diseases [13]. A20 negatively regulates the NF-κB signaling, a critical modulator of immunity, stress responses, cell proliferation and apoptosis [14]. Furthermore, the NF-κB signaling participates in the inflammatory response and behavioral manifestation of thermal hyperalgesia in peripheral nerve injury [15]. However, the role of neuron-derived EVs in SNI-induced neuropathic pain and associated inflammatory response remains poorly understood. Thus, in the present study, we investigate whether miR-23a delivered by DRG neuron-derived EVs contributes to inflammatory response and M1 polarization of macrophages in SNI models of neuropathic pain and examined A20 and NF-κB signaling as the possible mechanistic downstream target.

## RESULTS

### miR-23a was upregulated in DRG neurons derived from mice after SNI

**Figure 1:**
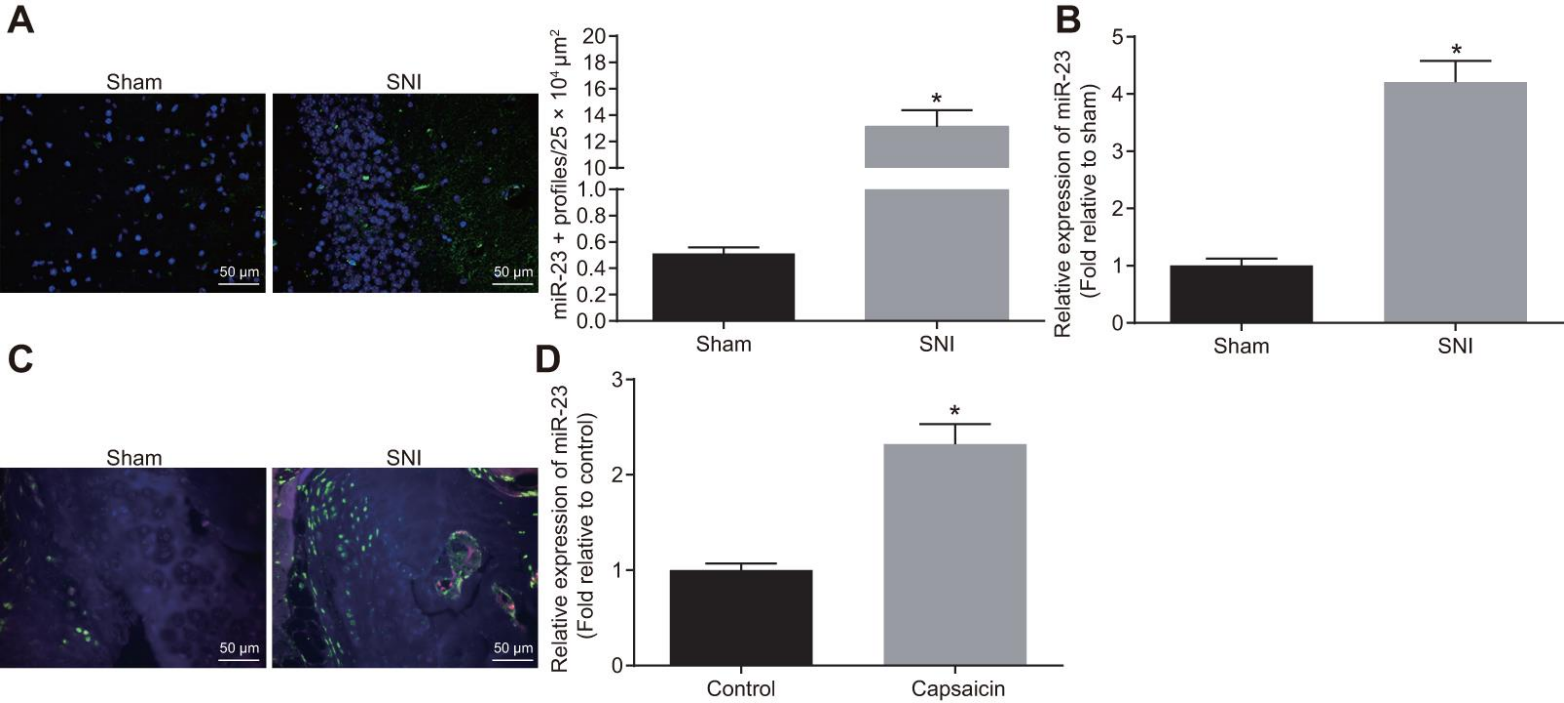
**miR-23a is upregulated in DRG neurons derived from mice after SNI.** (**A**) miR-23a expression in contralateral L5 DRG neurons at 7 day after model establishment, normalized to U6 (Scale bar = 50 μm) and quantitative histogram of miR-23a + neurons using FISH; (**B**) miR-23a expression in DRG neurons of sham-operated and SNI mice were determined using RT-qPCR, normalized to U6; (**C**) macrophages in DRG (F4/80+ cells, red) and miR-23a (green) detected using FISH and immunohistochemistry (Scale bar = 50 μm); (**D**) miR-23a expression in DRG neurons treated with 0.001% DMSO in HEPES buffer + glucose (1 mg/mL) and with capsaicin (1 μM), normalized to U6. Values obtained from three independent experiments are expressed as mean ± SD and analyzed by unpaired *t*-test. * indicates *p* < 0.05. Cell experiment was independently repeated for three times.

The miR-23a expression was analyzed using fluorescence *in situ* hybridization (FISH) and reverse transcription quantitative polymerase chain reaction (RT-qPCR) in lumbar vertebra DRG and DRG neurons of sham-operated and SNI mice. Compared with the sham-operated mice, SNI mice exhibited drastically increased expression of miR-23a (*p* < 0.05) (Figure 1A, 1B). Immunohistochemistry analysis revealed that compared to F4/80+ cells surrounding DRG of sham-operated mice, the number of macrophages near the ipsilateral DRG was increased significantly in SNI mice (*p* < 0.05) (Figure 1C). In a chemical-induced *in vitro* injury model, DRG neurons were treated with 1 μM capsaicin for 5 h, and the miR-23a expression was evaluated by RT-qPCR. Similarly, capsaicin treatment elevated miR-23a expression in DRG neurons (*p* < 0.05) (Figure 1D). Overall, miR-23a expression was elevated in injured DRG neurons and was associated with the increased number of macrophages *in vivo*.

### EVs-encapsulated miR-23a was enriched in cultured DRG neurons following capsaicin induction

**Figure 2:**
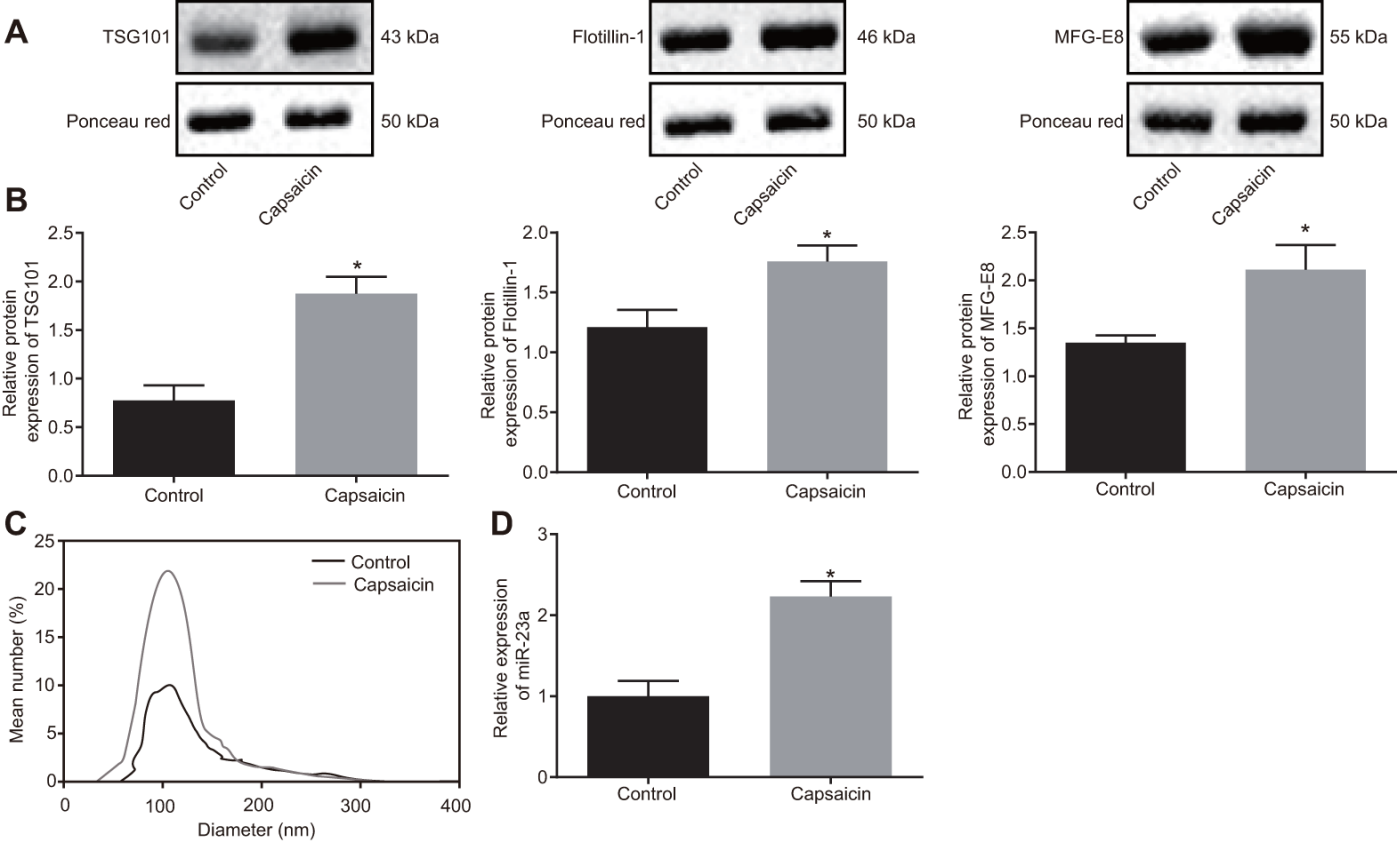
**EVs-encapsulated miR-23a is enriched in cultured DRG neurons following capsaicin induction.** (**A**) representative Western blots of TSG101, Flotillin-1, and MFG-E8 proteins in DRG neurons treated with capsaicin, normalized to β-actin; (**B**) quantitation of the protein levels of TSG101, Flotillin-1, and MFG-E8 in DRG neurons treated with capsaicin measured using Western blot analysis, normalized to β-actin; (**C**) EVs detected by NanoSight; (**D**) miR-23a expression in DRG neurons treated with capsaicin determined using RT-qPCR, normalized to U6. Values obtained from three independent experiments are expressed as mean ± SD and analyzed by unpaired *t*-test. * indicates *p* < 0.05. Cell experiment was independently repeated for three times.

In order to investigate whether neurons enhance the secretion of miRNA-containing EVs after noxious-like activation, DRG neurons were incubated with capsaicin (1 μM) for 5 h. The results showed that the expression of TSG101 and Flotillin-1 was increased (*p* < 0.05) (Figure 2A). In addition, MFG-E8 protein level was also elevated in DRG neurons treated with capsaicin (*p* < 0.05) (Figure 2B). Nanoparticle tracking analysis (NTA) confirmed that after treatment with capsaicin, the number of secreted EVs was increased to 1 fold, with enriched with EVs in either control or capsaicin-treated DRG neurons (Figure 2C). RT-qPCR presented that miR-23a was highly expressed in EVs in capsaicin-treated DRG neurons (*p* < 0.05) (Figure 2D). The obtained data suggested that capsaicin could induce DRG to secrete EVs-encapsulated miR-23a.

### DRG sensory neurons transferred miR-23a to macrophages *via* EVs to enhance M1 polarization *in vitro*

**Figure 3:**
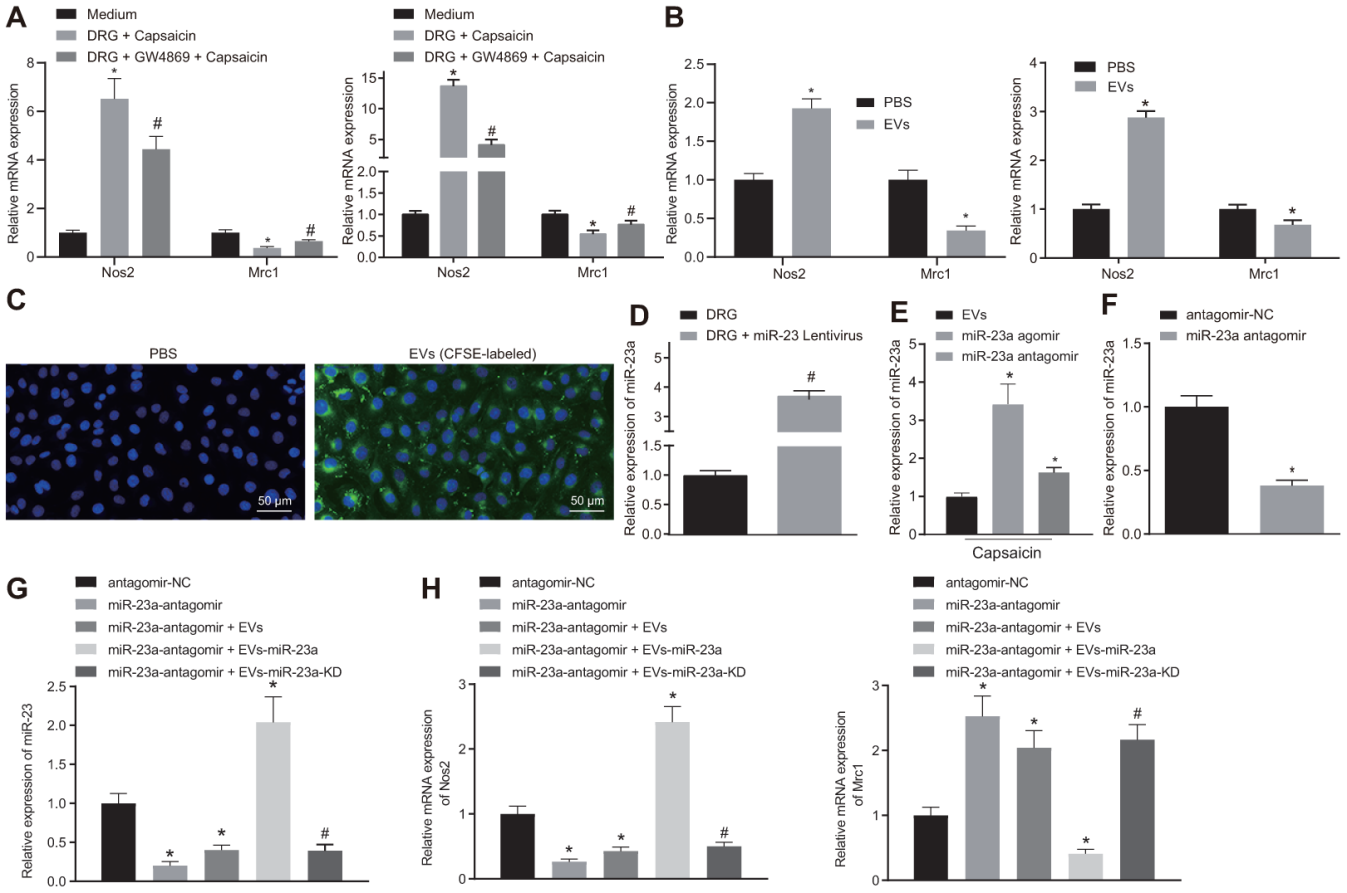
**DRG sensory neurons transferred miR-23a to macrophages *via* EVs to enhance M1 polarization *in vitro*.** (**A**) mRNA expression of Nos2 (M1) and Mrc1 (M2) in macrophages co-cultured with DRG neurons and treated with EV release inhibitor GW4869 (RT-qPCR, normalized to U6); (**B**) mRNA expression of Nos2 (M1) and Mrc1 (M2) in macrophages treated with DRG-secreted EVs induced by capsaicin determined using RT-qPCR, normalized to β-actin; (**C**) DRG neuron-derived EVs in macrophages observed using immunofluorescence staining (Scale bar = 50 μm); (**D**) miR-23a expression in DRG neurons treated with lentivirus-overexpressing miR-23a determined using RT-qPCR, normalized to U6; (**E**) miR-23a expression in DRG neurons treated with lentivirus-overexpressing miR-23a following capsaicin treatment determined using RT-qPCR, normalized to U6; (**F**) miR-23a expression in macrophages determined using RT-qPCR, normalized to U6; (**G**) miR-23a expression in macrophages with miR-23a knockdown and then treated with EVs determined using RT-qPCR, normalized to U6; (**H**) mRNA expression of Nos2 (M1) and Mrc1 (M2) in macrophages with miR-23a knockdown and then treated with EVs determined using RT-qPCR, normalized to β-actin. Values obtained from three independent experiments are expressed as mean ± SD and analyzed by unpaired *t*-test between two groups and by one-way ANOVA, followed by Bonferroni’s multiple comparison test among multiple groups. * *p* < 0.05 *vs.* apical chamber in transwell added with F-12 medium, DRG neurons, or DRG neurons treated with antagomir-NC plasmids; # *p* < 0.05 *vs.* DRG neurons, or DRG neurons treated with antagomir-NC + EVs-miR-23a. Cell experiment was independently repeated for three times.

Since both miR-23a and macrophages are enriched in DRG area in mice after SNI, we hypothesized that DRG neurons may communicate with macrophages through released miR-23a-containing EVs. To test this hypothesis, we co-cultured DRG neurons with mouse macrophages using an *in vitro* Transwell system and added capsaicin for treatment. When co-cultured with DRG neurons, macrophages showed increased Nos2 (M1) mRNA expression yet decreased Mrc1 (M2) mRNA expression, suggesting M1 polarization of macrophages. Such induction of polarization was abolished by an EV release inhibitor GW4869 (*p* < 0.05) (Figure 3A). These findings implied that EVs released by DRG promoted M1 polarization of macrophages. Similar results were observed when macrophages were treated directly with isolated EVs with more obvious trend noted upon capsaicin treatment (Figure 3B). To further verify that DRG neuron-derived EVs are phagocytosed by macrophages, we labeled the DRG neuron-derived EVs with carboxyfluorescein diacetate succinamidyl ester (CFSE, green fluorescence) and co-cultured them with macrophages for 1 h. Indeed, fluorescence microscopy imaging showed that a strong green fluorescent protein (GFP) fluorescence signal present within macrophages, suggesting the uptake of DRG neuron-derived EVs (Figure 3C). Moreover, DRG neurons were treated with lentivirus expressing miR-23a or miR-23a antagomir (Figure 3D) and then the EVs were isolated. miR-23a level was upregulated in the EVs derived from DRG neurons infected with lentivirus expressing miR-23a, which was negated following miR-23a antagomir treatment (*p* < 0.05) (Figure 3E). In addition, we treated macrophages with miR-23a antagomir (1 μg) (Figure 3F) and then treated miR-23a antagomir-treated macrophages with EVs. The results showed that the expression of miR-23a was increased in EVs-treated macrophages transfected with miR-23a (Figure 3G), in addition to increased Nos2 (M1) mRNA expression and decreased Mrc1 (M2) mRNA expression (*p* < 0.05) (Figure 3H). However, miR-23a antagomir treatment resulted in reduced miR-23a and Nos2 expression yet elevated Mrc1 expression in EVs-treated macrophages. The above findings elucidated that DRG neurons could promote a pro-inflammatory phenotype in macrophages *via* miR-23a, which are delivered by EVs *in vitro*.

### A20 was a target gene of miR-23a

**Figure 4:**
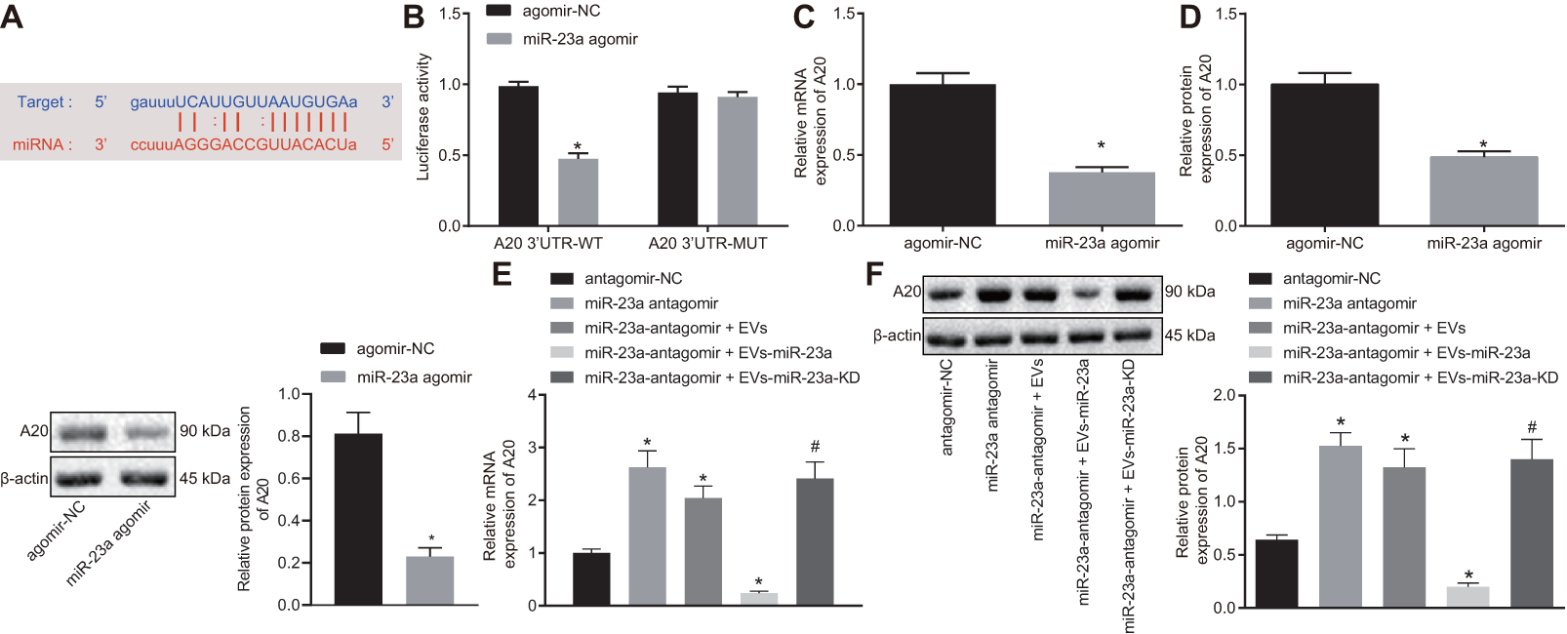
**miR-23a targets and negatively regulates A20.** (**A**) predicted binding sites between miR-23a and A20 mRNA 3’-UTR; (**B**) detection of luciferase activity using dual-luciferase reporter assay; (**C**) A20 mRNA expression in macrophages after elevated expression of miR-185 by its specific agomir determined using RT-qPCR, normalized to β-actin; (**D**) A20 protein level in macrophages after elevated expression of miR-185 by its specific agomir determined using Western blot analysis, normalized to β-actin; (**E**) A20 mRNA expression in macrophages co-cultured with EVs determined using RT-qPCR, normalized to β-actin; (**F**) A20 protein level in macrophages co-cultured with EVs determined using Western blot analysis, normalized to β-actin. Values obtained from three independent experiments are expressed as mean ± SD and analyzed by unpaired *t*-test between two groups and by one-way ANOVA, followed by Bonferroni’s multiple comparison test among multiple groups; n = 3 cultures. * *p* < 0.05 *vs.* macrophages treated with antagomir-NC plasmids; # *p* < 0.05 *vs.* macrophages treated with antagomir-NC + miR-23a expression EVs. Cell experiment was independently repeated for three times.

mmu-miR-23a binding sites were found in the 3’-UTR of A20 mRNA (Figure 4A). To further verify whether A20 is a target gene of miR-23a, we performed dual-luciferase reporter assay. Indeed, the luciferase activity of A20 3’-UTR-wild type (wt) was reduced in HEK293T cells in the presence of miR-23a agomir but that of A20 3’-UTR-mutant (Mut) showed no changes (Figure 4B). In addition, decreased A20 expression was observed in macrophages after elevating the expression of miR-23a using its specific agomir (*p* < 0.05) (Figure 4C, 4D). Moreover, A20 expression was also reduced in macrophages co-cultured with isolated EVs (*p* < 0.05) (Figure 4E, 4F). Thus, A20 was a direct target gene of miR-23a.

### Overexpression of miR-23a promoted M1 polarization of macrophages *via* activation of the NF-κB signaling by inhibiting A20 *in vitro*

**Figure 5:**
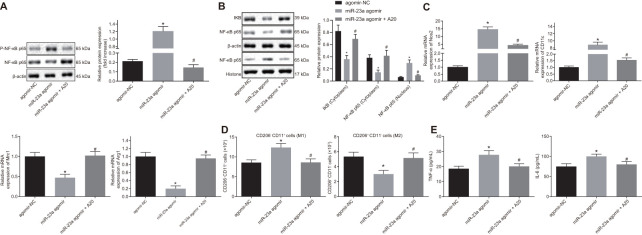
**Overexpression of miR-23a enhances inflammation response and M1 polarization of macrophages by inhibiting A20 and activating NF-κB.** Macrophages were treated with exogenous miR-23a agomir (agomir-NC used as control) or exogenous miR-23a agomir and A20. (**A**) protein levels of p-NF-κB p65/NF-κB p65 in macrophages measured using Western blot analysis, normalized to β-actin; (**B**) protein levels of NF-κB p65 in the nucleus and cytoplasm measured using Western blot analysis, normalized to β-actin; (**C**) mRNA expression of Nos2, CD11c, Mrc1, and Arg1 determined using RT-qPCR in macrophages, normalized to β-actin; (**D**) M1 polarization of macrophages detected by flow cytometry; (**E**) levels of TNF-α and IL-6 measured by ELISA in macrophages. Values obtained from three independent experiments are expressed as mean ± SD and analyzed by one-way ANOVA followed by Bonferroni’s multiple comparison test among multiple groups. * *p* < 0.05 *vs.* macrophages treated with antagomir-NC plasmids; # *p* < 0.05 *vs.* macrophages treated with miR-23a agomir plasmids. Cell experiment was independently repeated for three times.

In order to explore the role of miR-23a/A20 in the inflammatory response, macrophages were treated with exogenous miR-23a agomir with or without A20. Western blot analysis revealed an increase in the protein levels of phosphorylated (p)-NF-κB p65/NF-κB p65 along with NF-κB p65 expression in the nucleus yet a decrease in the expression of IκB and NF-κB p65 in the cytoplasm in the presence of miR-23a agomir. By contrast, protein levels of p-NF-κB p65/NF-κB p65 as well as NF-κB p65 expression in the nucleus were decreased while expression of IκB and NF-κB p65 in the cytoplasm was increased in the presence of miR-23a agomir and A20 (*p* < 0.05) (Figure 5A, 5B), suggesting that miR-23a activates the NF-κB signaling while A20 inhibits the signaling activation. Moreover, treatment of miR-23a resulted in increased Nos2 and CD11c transcription and reduced Mrc1 and Arg1 transcription, and treatment of both miR-23a and A20 partially or fully rescued the phenotype (*p* < 0.05) (Figure 5B). Flow cytometry also confirmed M2-to-M1 transition in macrophages by miR-23 treatment but not by combination treatment (Figure 5C). In addition, expression of tumor necrosis factor-α (TNF-α) and interleukin (IL)-6, evaluated by enzyme-linked immunosorbent assay (ELISA), was found to be increased in the presence of miR-23a agomir, but decreased in the presence of miR-23a agomir and A20 (*p* < 0.05) (Figure 5D). These results revealed that miR-23a may promote inflammatory response and M1 polarization of macrophages by inhibiting A20 and activating the NF-κB signaling *in vitro*.

### Inhibition of miR-23a delivery by DRG neuron-derived EVs repressed neuropathic hypersensitivity and M1 macrophages in mice following peripheral nerve injury

**Figure 6:**
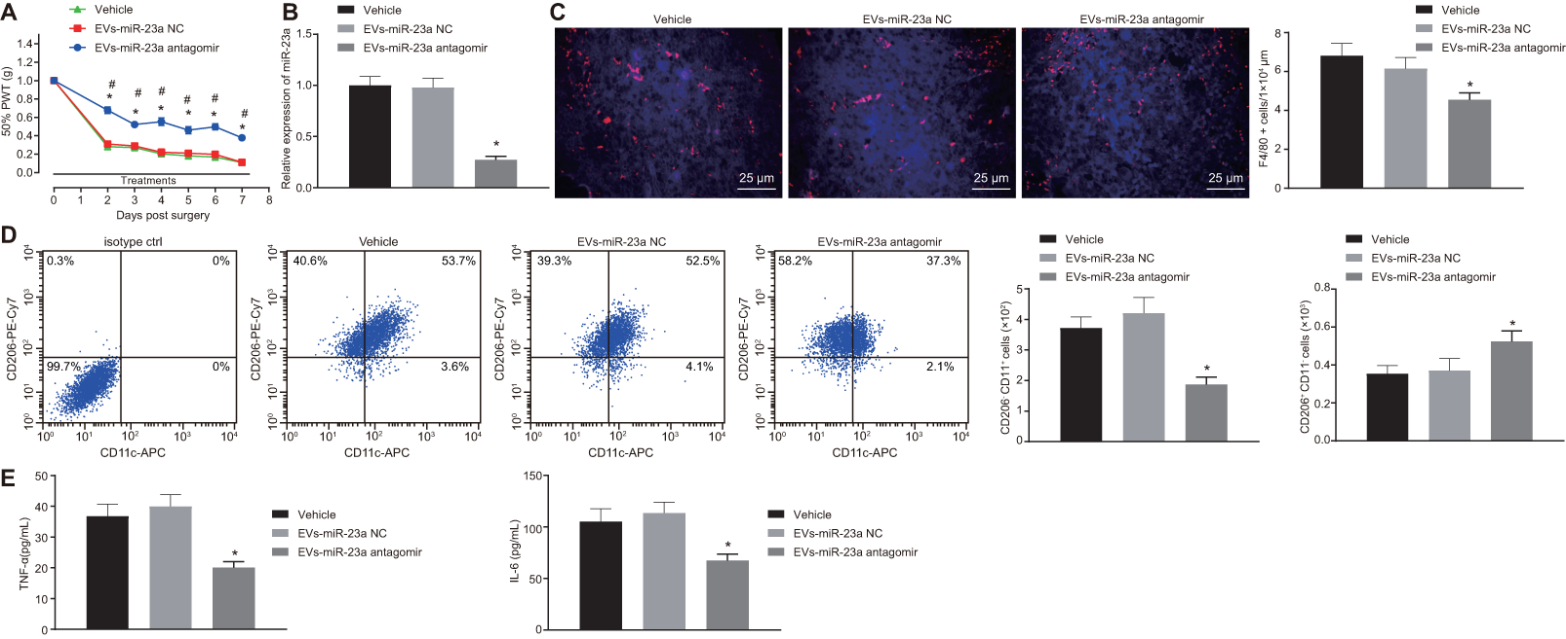
**Delivery of decreased miR-23a by DRG neuron-derived EVs reduces neuropathic hypersensitivity and recruitment of M1 macrophages *in vivo*.** SNI mice were treated with intrathecal administration of EVs-treated miR-23a antagomir-transfected DRG neurons. (**A**) effect of intrathecal injection of EVs-miR-23a antagomir on mechanical hypersensitivity for 7 days, which was expressed as mean ± SD of 50% PWT; n (vehicle mice) = 6, n (oligomer-treated mice) = 12; * *p* < 0.05 *vs.* vehicle mice; # *p* < 0.05 *vs.* oligomer-treated mice. Values obtained from three independent experiments in triplicate are expressed as mean ± SD and analyzed by two-way ANOVA, followed by Tukey’s test among multiple groups. (**B**) miR-23a expression in DRG determined using RT-qPCR, normalized to U6; (**C**) Immunostaining of macrophages (F4/80+ cells, red), and nuclei (DAPI, blue) in SNI DRG (scale bar = 25 μm); (**D**) Macrophage phenotyping [M1 macrophages (CD206^-^CD11c^+^) and M2 macrophages (CD206^+^CD11c^-^)] in DRG analyzed using flow cytometry; (**E**) levels of TNF-α and IL-6 in L4-L5 DRG tissues measured using ELISA. Values obtained from three independent experiments are expressed as mean ± SD and analyzed by unpaired *t*-test between two groups in panel (**B**–**E**). * *p* < 0.05 *vs.* mice treated with EVs-miR-23a-NC or vehicle. Cell experiment was independently repeated for three times.

Finally, we aimed to investigate the role of miR-23a delivered by DRG neuron-derived EVs in pain hypersensitivity and macrophages *in vivo.* We inhibited the expression of miR-23a in DRG neurons and collected the EVs in each group. The osmotic pump of normal saline or EVs (1.4 mg/mL at a dose of 1.4 mg per hour) was connected to the intrathecal catheter for 7 days. SIN mice were intrathecally administrated with EVs-miR-23a antagomir. RT-qPCR displayed that miR-23a expression was downregulated in DRG after intrathecal administration of EVs-miR-23a antagomir (*p* < 0.05) (Figure 6B), which confirmed the effective delivery of the construct. Compared to the control, intrathecal administration of EVs-miR-23a antagomir resulted in ameliorated pain hypersensitivity (Figure 6A). In addition, immunohistochemistry analysis revealed a decreased number of macrophages in ipsilateral DRG after intrathecal administration of EVs-miR-23a antagomir (*p* < 0.05) (Figure 6C). Flow cytometry displayed reduced M1 macrophages and increased M2 macrophages in ipsilateral DRG after intrathecal administration of EVs-miR-23a antagomir (*p* < 0.05) (Figure 6D). ELISA showed decreased levels of TNF-α and IL-6 in ipsilateral DRG after intrathecal administration of EVs-miR-23a antagomir (*p* < 0.05) (Figure 6E). The above findings elucidated that the delivery of reduced miR-23a by DRG neuron-derived EVs could reduce DRG neuropathic hypersensitivity and M1 macrophages following peripheral nerve injury *in vivo*.

## DISCUSSION

Neuropathic pain has severe adverse impacts on the quality of life of patients. There are few effective treatment options for neuropathic pain in the clinic, primarily due to its unknown mechanism of action [16]. EVs have demonstrated immunomodulatory and neuroprotective effects against nerve disorders as a cell free therapy [17]. Our study explored the effect of miR-23a-containing EVs from DRG neurons on inflammation and M1 polarization of macrophages in the SNI model of neuropathic pain.

First, we demonstrated that miR-23a was highly expressed in DRG neurons of SNI mice. As shown in a previous study, the dysregulation of miRNAs is related to the peripheral nerve injury of the primary sensory neurons [18]. Specific miRNA, such as miR-21, has been reported to be upregulated in the DRG of neuropathic rats [9]. Increased miR-23a expression is involved in brain maturation [11]. Moreover, the present study displayed that A20 is a key target gene of miR-23a. A20 plays an essential role in various immune cell functions and many experimental diseases. A previous study has confirmed that A20 is one of the target genes of miR-23a [12], further supporting the relationship between miR-23a and A20.

Second, our data demonstrated that upon injury, DRG neurons produced more miR-23a and delivered miR-23a to macrophages *via* EV release. A recent publication indicated that in the DRG, neuro-immune interaction post peripheral nerve injury is accomplished through the EV release by neurons, which are engulfed by nearby macrophages; the EVs deliver several determinants including miRNAs, with the potential to afford long-term alterations in macrophages affecting pain mechanisms [19]. Moreover, DRG neuron-derived EVs induced by capsaicin have been demonstrated to be able to transfer miR-21-5p to macrophages to promote inflammation in nerve injury [20], which supports our findings that DRG neurons elevated miR-21-5p and delivered it to macrophages through EVs.

Third, the present study showed that miR-23a transferred by DRG neuron-derived EVs promoted inflammatory response and M1 polarization of macrophages by inhibiting A20 and activating the NF-κB signaling. While miR-23a downregulation could inhibit pain hypersensitivity in DRG neurons of SNI models of neuropathic pain. It is known that inflammation and nerve damage could result in pain hypersensitivity to non-noxious stimuli injury [2]. A recent study has revealed that M1 activation is associated with increased pro-inflammatory factors, such as TNF-α, which elevates the sensitivity of somatosensory neurons and enhances neuropathic pain [4]. It is interesting to note that repression of miR-21 could ameliorate neuropathic pain behavior [9]. Simeoli et al. have implied that miR-21-5p expression is elevated in DRG neurons and downregulation of miR-21 in sensory neurons can decrease neuropathic hypersensitivity and inflammatory macrophage recruitment in the DRG after nerve injury [20]. Also, miR-23a has been revealed to play a vital role in macrophage polarization; for instance, overexpression of miR-23a enhances the activation of the NF-κB signaling by targeting A20 and thus promotes inflammatory responses [12], while silencing of miR-23a exerts an inhibitory effect on inflammation. Furthermore, the inhibition of NF-κB attenuates pain and inhibits inflammation after peripheral nerve injury, suggesting that targeting the inflammatory response may be an effective approach in treating neuropathic pain [21]. Herein, it is reasonable that DRG neurons delivering miR-23a are possessed with a great therapeutic potential in inflammation response and M1 polarization of macrophages in SNI-induced NP.

**Figure 7:**
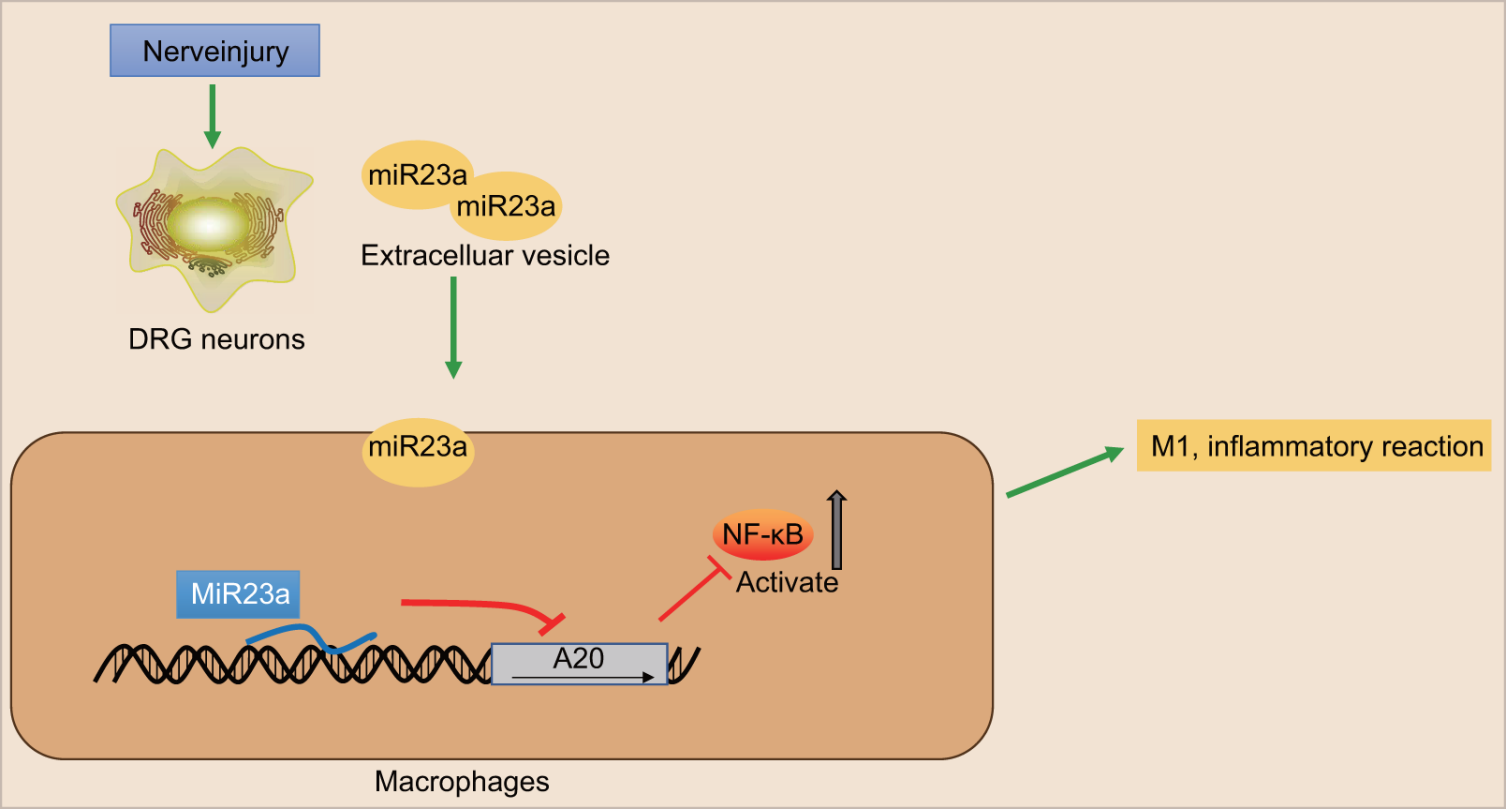
**The scheme illustrates the mechanism of action of miR-23a in the nerve injury and pain initiation.** Sensory neuron-derived EVs-encapsulated miR-23a exacerbates neuropathic pain and promotes M1 polarization of macrophages in peripheral nerve injury by inhibiting A20 and activating the NF-κB signaling.

In conclusion, we have proved that secreted EVs from DRG neurons contain miR-23a and are taken up into macrophages, thereby promoting inflammation and M1 polarization of macrophages by inhibiting A20 and activating the NF-κB signaling (illustrated in Figure 7). Thus, targeting the production or secretion of miR-23-containing EVs from DRG neurons may be a promising approach for developing novel therapeutic options for SNI-induced neuropathic pain. However, due to EVs rich in many substances, there may be other regulatory axes that affect the peripheral nerve injury, and further investigations are still needed to fully understand the mechanisms.

## MATERIALS AND METHODS

### Experimental animals

A total of 48 healthy male C57BL/6J mice (aged 8 weeks) were purchased from Dashuo Laboratory Animal Technology Co. (Chengdu, China). The mice were housed in an environment with a 12-h light/dark cycle and fed with standard diet. The mice underwent at least one-week adaptive feeding before the experiment. Six mice were treated with sham operation, and the remaining mice were used to establish mouse models. The success rate of model establishment was 85.7%, with a total of 36 mice successfully modeled. Next, these 36 mice were divided into the SNI group (n = 6), vehicle group (n = 6), and EVs-treated group (n = 24) which consisted of the EVs-miR-23a-negative control (NC) group (n = 12) and the EVs-miR-23a antagomir group (n = 12).

### Establishment of SNI mouse models

SNI and sham-operated mice were anesthetized with 1% pentobarbital sodium (50 mg/kg i.p.). For SNI mice, after exposure of the sciatic nerve and its three peripheral branches, the axons of the common tibiofibular branch were excised and ligated to preserve the integrity of the sural nerve. Muscle and skin layers were carefully sutured. For sham-operated mice, the sciatic nerve was exposed without axon incision and ligation, and muscle and skin layers were then carefully sutured. The complete sural nerve should not be stretched during the operation [22].

### Behavioral testing

The calibrated von Frey monofilaments were applied (0.02-1.0 g) to the hind paw plantar surface to assess the static mechanical withdrawal thresholds. Evaluation started with the application of a 0.07 g filament and each paw was assessed alternately between application of increasing stimulus intensity until a withdrawal response was achieved or application of 1.0 g filament failed to induce a response, in order to avoid tissue injury. The 50% of paw withdrawal threshold (PWT) was determined by increasing or decreasing stimulus intensity and evaluated using Dixon’s “up-down” method. The experiments were performed by blinded investigators.

### Intrathecal delivery of oligomers

Intrathecal cannula catheterization was performed on the same day of SNI surgery. After anesthesia, a small laminectomy was made over the thoracic spinal cord. A polyethylene catheter (Alzet, Charles River Ltd, UK) was inserted under the dura mater in the lumbar enlargement and attached to a subcutaneous osmotic pump (Alzet 1007D, Charles River Ltd). The locked nucleic acid (LNA)-based miR-23a inhibitor and interfered control oligomer were custom-made as fluorescein amidite (FAM)-labeled compounds by Exiqon (Copenhagen, Hovedstaden, Denmark). The oligomers were mixed (1:5 v/v) with i-Fect™ *in vivo* transfection reagent (Neuromics, 2B Scientific, UK) and delivered for 7 days *via* an osmotic pump at a rate of 12 pmol/day.

### FISH and immunohistochemistry

The cryostat (Thermo Fisher Scientific Shandon Cryotome FE, Thermo Fisher Scientific, Rockford, USA) was used to obtain the sections (10 μm) from freshly frozen L4 and L5 DRG, after which the sections were fixed on the glass slide of Superflost Plus (Thermo Fisher Scientific, Rockford, IL, USA). The frozen sections were then fixed in 4% paraformaldehyde and treated with Protease K (5 μg/ml, Sigma-Aldrich Chemical Company, St Louis, MO, USA). After acetylation with 1.35% triethanolamine/0.25% acetic anhydride/0.18% hydrochloric acid, the sections were pre-hybridized with 50% formamide, 5× saline sodium citrate, 0.5 mg/ml yeast tRNA and 1 × Denhardt’s solution. Next, the sections were hybridized with murine miR-23a complementary digoxigenin (DIG)-labeled probes (Roche Diagnostics GmbH, Mannheim, Germany) with scrambled probes as control. After hybridization, the sections were incubated with mouse anti-DIG horseradish peroxidase (HRP) antibody (1: 500, ab6212, Abcam Inc., Cambridge, MA, USA). *In situ* hybridization signaling was enhanced by the Alexa-488-labeled Tyramide amplification system (Invitrogen, Carlsbad, California, USA). For the subsequent immunohistochemistry, the sections were incubated with rabbit anti-mouse F4/80 (MA1-91124, 1: 1000, Invitrogen, Carlsbad, California, USA) and the secondary antibody alexa-568-labeled goat anti-rabbit F4/80 (A-11077, 1: 1000, Invitrogen, Carlsbad, California, USA) and were cover-slipped by Vectashield mounting medium with 4',6-diamidino-2-phenylindole (DAPI) (Vector Labs, Burlingame, CA, USA).

### Isolation and culture of primary DRG sensory neurons

DRG neuron was collected and placed into F-12 Nutrient Mixture (Ham; Gibco, Carlsbad, California, USA) containing 0.1% type IV collagenase (Worthington). After that, DRG neuron was triturated and the suspended cells were centrifuged at 600 rpm for 6 min. The precipitates were then resuspended in fresh DRG medium and added with nerve growth factor (NGF) (10 ng/mL; Promega, Madison, WI, USA), which was next plated on the coverslip pre-coated by poly-L-ornithine (100 μg/mL, Sigma-Aldrich Chemical Company, St Louis, MO, USA) for incubation (10,000-22,500 cells/well) at 37° C for 24 h. In the experiments where miR-23a was overexpressed, cultured neurons were transduced with a green fluorescent protein (control) or miR-23a lentiviral vector and were incubated at 37° C for 72 h. The purified primary DRG neurons were obtained using anti-biotin microbeads and magnetic activated cell sorting (MACS) technology (Miltenyi Biotec, Bergisch Gladbach, Germany). DRG neurons were transfected with miR-23a antagomir (1 μg) and the corresponding controls (Shanghai GenePharma Co., Ltd., Shanghai, China). On the experimental day, neurons underwent incubation with 0.001% dimethyl sulphoxide (DMSO) or 1 μM capsaicin (Capsaicin is a selective sensory nerve blocker, which can specifically act on the sensory nerve and stimulate the excitation of primary neurons *in vitro*. Therefore, capsaicin is an important tool for pain research *in vitro* and *in vivo* [23]) for 5 h. After stimulation, an aliquot of the supernatant was collected and EVs were then isolated by ultracentrifugation. The cells were collected for western blot analysis and RT-qPCR detection.

### Macrophage treatment

Macrophages were obtained by lavage of the peritoneal cavity with 1% penicillin/streptomycin sterile saline, plated, and allowed to adhere. Subsequently, non-adherent cells were removed by washing and adherent macrophages were covered with phenol-red-free complete Dulbecco’s modified Eagle’s medium (DMEM) (Gibco, Carlsbad, California, USA) supplemented with 10% heat-inactivated-fetal bovine serum (HI-FBS), 1% pen/strep, and 1% sodium pyruvate under 5% CO_2_ at 37° C. Adherent cells (1 × 10^6^ cells/well) were cultured at 37° C for 6 h and treated with miR-23a agomir, miR-23a antagomir (1 μg) or their corresponding controls. Cells treated with antagomir were cultured at 37° C for 48 h, and then treated with EVs. Macrophages receiving no treatment or treated with phosphate-buffered saline (PBS) were used as controls.

### RNA isolation and quantitation

For miRNA detection in EVs, EVs were isolated from the supernatant using the miRCURY™ Exosome Isolation Kit (Exiqon). The small RNA was purified using mirVana miRNA Isolation Kit (Ambion, Company, Austin, TX, USA). RT-qPCR for miR-23a was conducted, and miR-23a expression was analyzed by the 2^−ΔΔCT^ method with U6 as the internal control.

For miRNA and mRNA detection in cells, the total RNA was extracted using a Trizol kit (16096020, Thermo Fisher Scientific Inc., Waltham, Massachusetts, USA). Next, total RNA (5 μg) was reversely transcribed into complementary DNA (cDNA) according to the instructions of the cDNA kit (K1622; Fermentas Inc., Ontario, CA, USA). miR-23a expression was then determined using the TaqMan miRNA assay (Ambion, Austin, TX, USA) with U6 as the internal control. The PrimeScript RT-qPCR kit (TaKaRa, Shiga, Japan) was employed to measure the levels of A20, Nos2, Mrc1, CD11c, and Arg1 with β-actin as the internal control. The primer sequences are shown in Table 1. The expression ratio of target gene between the experimental and control groups was calculated using the 2^-ΔΔCt^ method.

**Table 1:** Primer sequences for RT-qPCR.

**Gene**	**Forward sequence**	**Reverse sequence**
miR-23a	5’-ATCACATTGCCAGGGATTTCC-3’	5’-CTCAACTGGTGTCGTGGA-3’
U6	5’-ATCGGTTGGCAAACGTTTC-3’	5’-TGCGAGTGGTTTTTGA-3’
A20	5’-CCTCTTCTTCGCCTGCTTTGTCC-3’	5’-CCCCGTCACCAAGCCGTTGTACC-3’
Nos2	5’-ACATCGACCCGTCCACAGTAT-3’	5’-CAGAGGGGTAGGCTTGTCTC-3’
Mrc1	5’-CTCTGTTCAGCTATTGGACGC-3’	5’-CCCCGTCACCAAGCCGTTGTACC-3’
CD11c	5’-CTGGATAGCCTTTCTTCTGCTG-3’	5’-GCACACTGTGTCCGAACTCA-3’
Arg1	5’-GCTCCAAGCCAAAGTCCTTAGAGAT-3’	5’-AGGAGCTGTCATTAGGGACATCAAC-3’
β-actin	5’-CTGTCCCTGTATGCCTCTG-3’	5’-ATGTCACGCACGATTTCC-3’

### Western blot analysis

For detection of EV markers in sensory neuron: DRG neuron-derived EVs were resuspended in cold lysis buffer (Tris-HCl, 20 mM, pH 7.5, NaF 10 mM, NaCl 150 mM, 1% Nonidet P-40, phenylmethylsulfonyl fluoride 1 mM, Na3VO4 1 mM) containing protease inhibitor cocktail tablets (Roche Diagnostics GmbH, Mannheim, Germany) at a concentration of 1.4 mg/mL. Lysates were dissolved in 3× Laemmli’s sample buffer, boiled for 5 min, subjected to 12% sodium dodecyl sulfate-polyacrylamide gel electrophoresis (SDS-PAGE), and then transferred onto the nitrocellulose membrane. The membranes were blocked with 5% non-fat emulsion, PBS at pH 7.4 (137 mmol/L NaCl, 2.7 mmol/L KCl, 10 mmol/L Na_2_HPO_4_, and 2 mmol/L KH_2_PO_4_ and 0.05% Tween. After that, the membranes were probed overnight with rabbit anti-TSG101 (ab30871, 1:1000; Abcam Inc., Cambridge, UK), rabbit anti-Flotillin-1 (ab41927, 1: 1000; Abcam Inc., Cambridge, UK), and rabbit anti-MFG-E8 (1: 1000, Cat# sc-33546, Santa Cruz Biotechnology, Inc., Santa Cruz, CA, USA). Subsequently, the membranes were re-probed with HRP-labeled secondary antibody (Ab6721, 1: 10000; Abcam Inc., Cambridge, UK). Enhanced Chemiluminescence Detection System was utilized to detect protein signal (170-8280, Bio-Rad Laboratories Inc.; Hercules, CA, USA). Ponceau red was served as internal control. The data were analyzed using Image J (National Institutes of Health Inc., Bethesda, MD, USA).

Cell extracts: macrophages were lysed, and protein concentration was determined by Bradford assay (Bio-Rad, Richmond, Cal., USA) prior to gel electrophoresis on 10% SDS-PAGE, and transferred onto the nitrocellulose membrane. The membrane was probed with the following primary antibodies: IκB (4812, 1:1000, Cell Signaling Technology, Beverly, MA, USA), rabbit anti-p-NF-κB p65 (3033, 1: 1000; Cell Signaling Technology, Beverly, MA, USA), rabbit anti-NF-κB p65 (8242, 1: 1000, Cell Signaling Technology, Beverly, MA, USA), and rabbit anti-A20 (1: 500, ab74037, Abcam Inc., Cambridge, MA, USA). The β-actin (8457, 1: 1000, Cell Signaling Technology, Beverly, MA, USA) was used as internal reference and Histone H3 (4499, 1:1000, Cell Signaling Technology, Beverly, MA, USA) as intra-nuclear reference.

### EV isolation and analysis

Cell culture supernatant was collected into a 50 mL polypropylene test tube, which was then centrifuged at 500 ×g for 30 min and at 2000 ×g for 20 min at 4° C, with the supernatant collected. Obtained supernatant was treated with ultracentrifugation at 10000 ×g for 45 min and at 100000 ×g for 90 min. The precipitation was the EVs. EV size distribution analysis was performed using an NS300 Nanoparticle Tracker with 488 nm scatter laser and high-sensitivity camera (Malvern Instruments Ltd., Malvern, UK). For each sample, particle scatter was recorded three times for 60 s each under flow conditions (arbitrary speed 50) at camera level 16 and analysis threshold 5, using the NTA 3.2 acquisition and analysis software. For internalization of EVs by macrophages, EVs were stained with CellTrace™ CFSE (1:1000, Invitrogen, Carlsbad, California, USA) then ultracentrifuged at 100000 ×g for 1 h. Mouse macrophages (2 × 10^6^ cells/well) were incubated at 37° C for 24 h, and subsequently incubated with DRG neuron-derived EVs (100 μL) stained by CFSE for 1 h. Finally, the uptake of EVs (1.2 mg/mL) by macrophages was observed under a fluorescence microscope.

### Flow cytometry

Isolated DRG neurons and peritoneal macrophages were resuspended in Hank’s balanced salt solution (HBSS) supplemented with 1.5% bovine serum albumin (BSA). An aliquot of cell suspension was used for counting to obtain the absolute number of cells in each sample. Cells were stained on ice for 20 min with anti-mouse CD16/CD32 (Clone 2.4G2, BD Biosciences, Franklin Lakes, NJ, USA) to block Fc receptors, followed by incubation with fluorochrome-conjugated anti-mouse antibodies: rat monoclonal IgG2b antibody CD45.1-Pacific Blue™ (Clone 30-F11, BioLegend, San Diego, CA, USA), rat monoclonal IgG2a antibody F4/80-PE (Clone BM8, eBioscience, San Diego, CA, USA), rat monoclonal IgG2b antibody CD11b-APC (Clone M1/70, eBioscience), rat monoclonal IgG2a antibody CD206-PE-Cy7 (Clone C068C2, BioLegend, San Diego, CA, USA), and hamster (Armenian) monoclonal IgG antibody CD11c-APC eFluor780® (Clone N418, eBioscience, San Diego, CA, USA). Isotope IgG antibody of the same concentration was added as the NC group After washing, cells were diluted in flow buffer and run through a LSRFortessa™ cell analyzer (BD Biosciences, Franklin Lakes, NJ, USA). Samples were finally analyzed with FlowJo software (Tree Start, Ashland, OR, USA).

### Dual-luciferase reporter assay

The binding between miR-23a and A20 was verified by dual-luciferase reporter assay. The artificially synthesized A20 3’-UTR gene fragments were introduced into pMIR-reporter using endonuclease sites SpeI and Hind III (Beijing Huayueyang Biotechnology Co., Ltd., Beijing, China). The complementary sequence mutation sites of seed sequences were designed on A20-wt. The target fragments were then inserted into the pMIR-reporter reporter plasmid using T4 DNA ligase after restriction endonuclease digestion. The correctly sequenced luciferase reporter plasmids of wt and mut were co-transfected with agomir-NC and miR-23a agomir plasmids into HEK293T cells (CRL-1415, Shanghai Xin Yu Biotech Co., Ltd., Shanghai, China), respectively. Following 48-h transfection, the cells were lysed. The luciferase activity was then detected using the Dual Luciferase Reporter Assay Kit (RG005, Beyotime Institute of Biotechnology, Shanghai, China) and Glomax20/20 luminometer (Promega Corporation, Madison, WI, USA), normalized to renilla luciferase. The relative luciferase unit (RLU) activity was calculated as the RLU activity of firefly luciferase/RLU activity of renilla luciferase.

### ELISA

The ELISA kits (MTA00B or M6000B, R&D Systems, Minneapolis, MN, USA) were used to measure the levels of TNF-α and IL-6 (pg/mL) in cell supernatant. The IL-6 content (pg/mL) in DRG tissue lysate was determined using ELISA kits (100712, Abcam Inc., Cambridge, MA, USA), and TNF-α content (pg/mL) using ELISA kits (SBJ-M0030, Nanjing SenBeiJia Biological Technology Co., Ltd., Nanjing, China).

### Statistical analysis

Statistical analysis was performed with SPSS 21.0 statistical software (IBM Corp. Armonk, NY, USA). All measurement data were expressed as mean ± standard deviation (SD). Comparisons of data conforming to the normal distribution and homogeneity of variance between two groups were conducted using unpaired *t*-test. Comparisons among multiple groups were analyzed using one-way analysis of variance (ANOVA), followed by Bonferroni’s multiple comparison test or two-way ANOVA, followed by Tukey’s test for behavioral data. The rank-sum (non-parametric) test was used for data with skewed distribution and unequal variances. A value of *p* < 0.05 was considered to be indicative of statistical significance.

### Ethics statement

The protocols were approved by the Animal Ethics Committees of Chengdu University. All animal experiments conducted during this study were in strict adherence with the Guide for the Care and Use of Laboratory Animals of the US National Institutes of Health. Measures were taken to minimize the usage of animals as well as their suffering.
